# Active Lives South Australia health economic analysis: an evidence base for the potential of health promotion strategies supporting physical activity guidelines to reduce public health costs while improving wellbeing

**DOI:** 10.1007/s10389-021-01649-0

**Published:** 2021-09-19

**Authors:** Simon Eckermann, Andrew R. Willan

**Affiliations:** 1 School of Health and Society, Faculty of Arts, Social Sciences and Humanities, University of Wollongong, Room 119, Building 29, Main Campus UOW Keiraville, Wollongong, NSW 2522 Australia; 2 Division of Biostatistics, Dalla Lana School of Public Health, University of Toronto, Toronto, ON Canada

**Keywords:** Physical activity guidelines, Subjective wellbeing, Health service use and cost, Health promotion

## Abstract

**Aim:**

The COVID-19 pandemic has threatened individual and population wellbeing and strategies to jointly address these challenges within budget constraints are required. The aim of our research is to analyse evidence from the Active Lives South Australia study to consider the potential of physical activity (PA) health promotion strategies to be health-system cost saving while addressing wellbeing challenges.

**Methods:**

The Active Lives South Australia study compares adult populations who meet and do not meet physical activity (PA) guidelines (150+ minutes of weekly physical activity) with respect to their subjective wellbeing and health care utilisation.

**Subject and results:**

Adults who met PA guidelines had better wellbeing across all aspects with and without adjustment for age, sex and income covariates. Analysis showed significant associations between meeting guidelines and lower probabilities of visiting and utilisation of GPs, specialist doctors, other health professionals, hospital inpatient admissions, outpatient clinic and emergency department visits, and an overall A$1760 lower cost per person annually. Controlling for age, sex and income, health expenditure for adults who met PA guidelines was significantly lower by A$1393 per person annually. That translated to A$804 million potential annual SA health system cost saving by shifting all adults to meeting PA guidelines.

**Conclusion:**

There is significant potential for effective health promotion strategies to be net cost saving while addressing wellbeing challenges of COVID-19 recovery where they can shift target populations from not meeting to meeting PA guidelines.

## Introduction

The underlying aim of this health economic analysis is to robustly estimate the potential for population level health system cost savings alongside wellbeing improvement from shifting adult populations from not meeting physical activity (PA) guidelines to meeting guidelines (World Health Organisation 2010), or more broadly towards meeting integrated movement guidelines (Ross et al. 2020). Such research is suggested to have particular value currently in informing budget constrained public policy need for effective health promotion strategies in coping with and recovering from the recent COVID-19 pandemic and development of Integrated Movement Guidelines (IMGs) in adults (18 up to 65 and 65 and older – Ross et al. 2020) as well as early childhood (0 up to 5 – Tremblay et al. 2017; Okely et al. 2017) and youth (5 up to 18 – Tremblay et al. 2016).

The Active Lives South Australia study survey undertaken in April and May 2019 across 2999 South Australian adults by computer-assisted telephone interview or online via phone or e-mail enables comparison of self-reported measures between adult populations who meet or do not meet physical activity guidelines, defined by whether they undertake 150 min or more of physical activity per week or not. Alongside physical activity questions the survey covered subjective wellbeing, individual development, community connectedness and social capital survey questions adapted from Sport England’s Active Lives Survey as well as health care access and utilisation. Analysis of results in the main report (Active Lives South Australia 2019) considered differences in self-reported survey questions across factors by self-reported physical activity per week (0, 1–149 min and 150 min or more), reported at a representatively age and sex weighted population level. That analysis found adult populations with higher levels of physical activity in meeting guidelines (150 or more minutes PA per week) relative to those not meeting guidelines (with 0 or 1–149 min PA per week) had associated improved wellbeing, individual development, social connectedness and social capital in analysis both without covariate adjustment and adjusting for age, sex and SEIFA index. Trends were also found for adult populations with 150 min or more PA having lower probability of visits in the last year to GP, specialist doctor or other health professional or hospital inpatient admissions, outpatient clinic and emergency department visits.

## Methods

To address the underlying aim of this report, three levels of increasingly robust and instructive sets of analyses using individual data are employed. The first set of analyses compare odds of individuals self-reported use of health care services (GP, specialist, other health professional, dental, hospital emergency department, inpatient and outpatient visits), levels of service use and associated expected cost to the health system in populations who meet and do not meet PA guidelines, using a gamma distribution for costs to account for positive skewing. It has been shown to be important with relative comparisons of binary variables (such as success or failure, the probability of visiting a health service provider or not) to use a symmetric metric such as the odds ratio to overcome problematic inconsistency with alternative framing of the same binary evidence comparing probabilities with non-symmetric metrics such as relative risk that arise in efficiency comparison (Eckermann et al. 2021) and/or evidence synthesis or translation (Eckermann et al. 2009, 2011; Eckermann 2017 Chap 3). While differences in relative chance of health service utilisation in populations meeting or not meeting PA guidelines are consistently established with ORs, implications for health system resource use and costs needs to consider mean differences in health service use and estimated associated costs across health service utilisation. Follow-up questions in the Active Lives survey asking about how many times in the past year participants used each service type enable overall yearly utilisation of resource use and costs across each of the major health service areas to be estimated (Active Lives South Australia 2019).

The second set of analyses considers physical activity as a continuous variable, again using individual respondent data and modelling cost data using a gamma distribution to account for positive skewing, to consider cost implications of marginal changes in population physical activity and consequently implications for the potential appropriate cut-point in the number of minutes of physical activity per week for meeting vs not meeting guidelines.

The third set of analyses jointly adjusts for significant age, sex and income covariates in comparisons between populations meeting and not meeting guidelines using individual data to estimate effects and cost differences under uncertainty and modelling cost data using a gamma distribution to account for positive skewing. To examine potential effects of confounding appropriately allowing for their multivariate interaction, regression models were used to estimate the difference in mean total cost between levels of physical activity (<150 vs ≥150 min/week) while jointly adjusting for sex, age and measures of socio-economic status. Because the measures of socio-economic status are highly correlated, income was entered into the regression models first, followed by each of the other measures (marital status, education, work status, home ownership). After entering sex, age and income into the regression models, each of the other measures of socio-economic status, entered one at a time, were not statistically significant and had no appreciable effect on the estimate on the parameters in the model, indicating that income alone was sufficient to adjust for socio-economic status. Hence, estimates and 95% confidence intervals for the difference in total costs between levels of physical activity are reported in multivariate analyses adjusting for all combinations of sex, age and income.

Comparison between these increasingly robust levels of analysis is instructive in seeing the importance and impact of allowing for covariate adjustment as well as comparing continuous vs dichotomous consideration of physical activity levels. Finally, sensitivity analyses are also undertaken to consider the impacts of removing influence of high cost respondents – those with greater than $75,000 health expenditure within 12 months.

### 1st analysis of proportional health service use for populations meeting vs not meeting PA guidelines (150+ vs 0–149 min PA/week)

For relative comparisons of binary variables, such as visiting a health service provider or not, use of odds ratios as a symmetric metric has been shown to overcome problems of non-symmetric metrics such as relative risk that arise with alternative framing of the same binary outcome (e.g. meet vs do not meet guidelines) in efficiency comparisons, evidence synthesis or translation (Eckermann et al. 2009, 2011, 2021; Eckermann 2017 chapter 3). We start by considering population odds ratios for having health service use across major categories in adult populations identified by individuals with 150+ minutes of physical activity (*n* = 1702) versus those with <150 min (*n* = 1294) across major categories of GP, specialist, other health professional, dental, hospital emergency department, inpatient and outpatient use in Table 1. That is, calculating the odds of visiting health services in these populations from their probabilities and subsequently the odds ratio (OR) between populations meeting PA guidelines (150+ min/week) and those not (0–149 min/week) is simply the ratio of these odds.^1^

**Table 1:** Probability, odds and odds ratio for health service use in active lives populations with 150+ vs 0–149 min physical activity per week

	Minutes of activity per week						OR	95% CI OR
	Less than 150 min (*N* = 1264)			150 min or more (*N* = 1702)				
	N	P (visit)	Odds visit	N	P (visit)	Odds visit		
GP	1216	0.947	17.868	1582	0.930	13.184	0.737	(0.543, 1.002)
Specialist doctor	753	0.586	1.418	874	0.514	1.055	0.744	(0.643, 0.862)*
Dentist	707	0.551	1.225	1132	0.665	1.986	1.621	(1.396, 1.881)*
Other health professional	542	0.422	0.730	694	0.408	0.689	0.943	(0.814, 1.092)
Hospital admission	341	0.266	0.362	342	0.201	0.251	0.695	(0.586, 0.825)*
Hospital outpatient clinic	302	0.235	0.308	283	0.166	0.199	0.648	(0.541, 0.777)*
Hospital ED	263	0.205	0.258	255	0.150	0.176	0.684	(0.566, 0.827)*

*Statistically significant with 5% type I error

The odds ratios and their 95% CIs in Table 1 show statistically significant lower relative odds of health service use in those meeting guidelines for each of specialist doctor (OR = 0.744), hospital inpatient admission (OR = 0.695), emergency department (OR = 0.684) and outpatient clinic services (OR = 0.648), while a statistically significant higher odds of using dental service (OR = 1.621). Trends towards reduction in GP use and other health professionals were also observed.

Symmetry of the odds ratio equivalently implies that the OR for not using services are simply their inverse or reciprocal of the OR for using services. That is, for the population meeting guidelines relative to that not meeting guidelines, the OR of not using specialist doctors is 1.34 = 1/0.744, not using hospital admission is 1.439 = 1/0.695, not using hospital emergency department presentations is 1.462 = 1/0.684 and not using outpatient services is 1.543 = 1/0.648. Similarly, by symmetry 95% confidence intervals for OR of not using services are the reciprocal of those for using services. Hence, the OR for not using services comparing populations meeting vs not meeting guidelines would equivalently and consistently be significantly greater than 1 for specialist, hospital inpatient admissions, emergency department and outpatient services and less than 1 for dental services. Consequently, odds ratios provide distinct advantages over relative risk where such symmetry does not arise in enabling consistent estimation with alternative framing for binary outcomes (Eckermann et al. 2009, 2011, 2021). Table 2 applies relevant average government scheduled fees given the mix of services across South Australian populations to the difference in service use per person–year between adult populations who meet and do not meet PA guidelines (have 150+ vs 0–149 min of PA per week) to estimate the difference in total cost per person–year.

**Table 2:** Average health care use and cost difference per person–year for 150+ vs 0–149 min of physical activity per week

	Mean utilisation with 0–149 min physical activity/week	Mean utilisation with 150+ min physical activity/week	Mean utilisation difference 150+ vs 0–149	Average scheduled fee per service (A$)	Mean cost difference per person-year (A$)	*p* value
GP*	7.639	4.974	−2.665	44.23	−117.88	<.0001
Specialist doctor*	2.458	1.722	−0.736	73.42	−54.07	<.0001
Dentist*	1.216	1.323	0.107	53.91	5.76	0.0889
Other health professional*	3.734	2.981	−0.752	57.75	−43.45	0.0088
Hospital admission^	0.504	0.307	−0.197	6053.00	−1193.63	<.0001
Hospital outpatient clinic^	0.974	0.437	−0.537	467.00	−250.86	<.0001
Hospital emergency department^	0.355	0.207	−0.148	715.00	−105.63	<.0001
Total government cost					−1760	<.0001

*SA adult price per MBS service type estimated from MBS data on the average cost of the service mix of relevant MBS scheduled fee items in SA populations 15 and over (i.e. 15–24 and all older age-groups) in the 2018/19 financial year and ABS (2019) SA populations by single year age groups

^Average adult price per hospital service calculated from SA Health Department data on non-paediatric hospital services in 2017/18 financial year (latest available data at time of analysis)

The direction of mean population health service utilisation in Table 2 generally mirrors that for the odds of using services in Table 1, while notably diminishing in extent for dental services particularly, given an OR of 1.621 while difference in average service use per year of less than 10% (1.323 vs 1.216). This arises because while populations who met PA guidelines had a significantly greater chance of using dental services, their net use of dental services was mitigated by lower average use of dental services when they did use them, which combined to result in a non-significant increase in mean use across the population (1.323 vs 1.216, *p* = 0.0889).

The population meeting PA guidelines with 150 min or more of weekly physical activity had significantly lower mean GP, specialist doctor, other health professional and hospital admissions, outpatient clinics and hospital emergence department service use and costs per year, while a non-significant trend towards overall greater mean dental service use and associated cost estimates.

Overall, for adult populations who meet versus do not meet physical activity guidelines (have 150 min or greater versus 149 min or less physical activity per week) combined net government health system costs of GP, specialist, other health professional, dentist and hospital inpatient admissions, ED and outpatient services are estimated given average service prices in SA for utilisation to be A$1760 lower per person–year. Given a current adult population in SA of 1,369,751, of which Active Lives survey findings estimate 42.12% do not meet PA guidelines the potential cost savings at a population level of health promotion and whole of government strategies in reducing net health system expenditure in SA is estimated at A$1015 million annually (Table 3).

**Table 3:** Potential for health system cost saving from adults meeting PA guidelines

SA adult population estimate June 2018	1,369,751
Survey population proportion not meeting guideline	42.12%
Estimated SA population not meeting guideline	576,954
Mean lower health cost per adult meeting vs not meeting PA guideline	A$1760
Potential annual lower mean net health care cost	A$1015 million

Hence, analysis employing individual data without covariate adjustment suggests if health promotion and whole of government strategies in support of PA guidelines enabled shifting SA adult populations who currently undertake 0–149 min of physical activity to have physical activity reflecting that of populations who undertake 150+ minutes, then net government health expenditure in SA has potential to reduce by A$1.015 billion annually. This would be attributable to an annual potential reduction of $688.8 million for hospital inpatient admissions, $60.9 million for hospital ED, A$144.7 million for hospital outpatients, A$68.0 million for cost of GP visits and A$31.2 million for specialist and other health professionals, while an increase in dental services of A$4.6 million (Table 4).

**Table 4:** Potential annual health system savings from all adults meeting PA guidelines by major area utilisation

	Estimated population not meeting PA	Cost saving per person–year (A$)	Total annual potential cost saving (A$ million)
GP*	576,954	−117.88	68.009
Specialist doctor*	576,954	−54.07	31.196
Dentist*	576,954	5.76	−3.323
Other health professional*	576,954	−43.45	25.071
Hospital admission^	576,954	−1193.63	688.668
Hospital outpatient clinic^	576,954	−250.86	144.733
Hospital ED^	576,954	−105.63	60.945
Total government cost		−1759.56	1015.3

*SA adult price per MBS service type estimated from MBS data on the average cost of the service mix of relevant MBS scheduled fee items in SA populations 15 and over (i.e. 15–24 and all older age-groups) in 2018/19 financial year

^Average adult price per hospital service calculated from SA Health Department data on non-paediatric hospital services in 2017/18 financial year (latest available data at time of analysis)

While this represents the potential cost saving from shifting the health care utilisation of the adult populations who do not meet PA guidelines (have 0–149 min per week of physical activity) to populations who do (have 150+ minutes PA per week), in reality, any individual strategy or set of strategies while aiming to have greatest incremental effects in improving PA and reducing healthcare costs don't expect to eliminate inactive populations. More generally, strategies might be expected to have intramarginal effects (within populations) in the population who may be amenable to change and/or inframarginal effects (across populations) of for example nudge strategies aimed at marginal impacts across whole populations.

To this end, below are considered the potential for cost savings from a series of potential 5% intra (shifting population) and/or infra (across population) marginal effects:

(i) An absolute 5% intramarginal reduction in the proportion of the population who do not meet PA guidelines, i.e. from 42.12% to 37.12%, which equates to potential cost savings of 0.05/0.4212 × A$1015 million = A$120.5 million per year.
(ii) A relative 5% intramarginal reduction in the proportion of the population who do not meet guidelines – from 42.12% to 40.06%, which equates to potential health system cost savings of 0.05 × 576,954 × A$1759.56 = A$50.8 million per year.
(iii) An inframarginal reduction in health care costs per person across the population who do not meet guidelines of 5% towards the level of that of populations who do meet guidelines which equates to potential health system cost savings of 0.05 × A$1015 million = A$50.8 million per year, the same as (ii).
(iv) A 5% inframarginal reduction in health care costs per person across the whole population who do not meet guidelines which equates to potential health system cost savings of 0.05 × A$4558.47 × 576,954 people = A$131.5 million per year.
(v) A 5% reduction in health care costs per person across the whole population which equates to potential cost savings of (iv) + 0.05 × $2798.71 × 792,797 people = A$131.5 million/year +A$110.9 million/year = A$242.4 million per year.

These are potential population level costs savings of marginal effects for one year noting that the Active Lives survey results are weighted to be representative of the South Australian adult population by age and sex. One could also consider the marginal effects from changing the physical activity trajectory of adult populations over a lifetime where the trajectory of marginal changes were maintained. Hence, for a representative adult across the population if they shifted at age 18 from what is observed in populations not meeting PA to meeting PA guidelines, they would be expected with life expectancy of 80 years to save lifetime undiscounted and unindexed health expenditure of: A$1759.56 per year × (80–18) years = A$109,104. That is an estimated A$109,104 undiscounted and health system cost saving across an adult lifetime form age 18 in changing the trajectory of physical activity from that of populations who do not meet to meet adult PA guidelines for 150+ minutes of physical activity a week. This lifetime estimate of government cost saving to the health system in SA of A$109,100 for an individual at age 18 from changing trajectory in shifting from not meeting to meeting PA guidelines is in line with the latest US estimates. The cost savings over a lifetime from 12 year-old children becoming physically active for cardiovascular, cancer and diabetes alone is estimated as USD 62,418 in a representative child, while more than USD 100,000 for populations with BMI of 30 or greater (Lee et al. 2017).

For a single year cohort of 21,259 SA 18-year-olds at June 302,018, an additional 5% or 1062 individuals positively changing the trajectory of their physical activity to meet PA guidelines, applying the lifetime estimate for health system cost savings of A$109,104 points to potential lifetime cost savings of A$115.9 million with sustained change. Changing the trajectory of PA by 5% for a generation (10 years) of such young adults would have expected cost savings in the order of tenfold this (that is, in the order of A$1.159 billion). This highlights the potential health system cost saving of strategies which have long term population level effects in changing trajectory of PA to meeting PA guidelines.

### 2nd analysis of PA as a continuous variable in informing marginal analysis

The second analysis uses individual respondent data and considers physical activity as a continuous variable to consider cost implications of marginal changes in population physical activity, the resulting dose effect and consequently implications for the potential appropriate cut-point in the number of minutes of physical activity per week for meeting vs not meeting guidelines. Using individual respondent data allows for modelling cost data using a gamma distribution to account for the positive skewing. The marginal changes in total health system cost with additional activity and ‘dose effect’ between minutes of physical activity and total cost is examined in Table 5.

**Table 5:** Number of respondents (N) and mean total cost by level of activity; and mean difference between adjacent levels

Minutes of activity/week	N	Mean (A$)	Lower limit (A$)	Upper limit (A$)	Marginal mean difference (A$)	Lower limit (A$)	Upper limit (A$)
0–29	601	5551	4880	6222	−1779	−2670	−887
30–89	363	3772	3186	4359	−181	−995	633
90–149	320	3591	3012	4170	−1201	−1930	−473
150–209	213	2390	1930	2851	464	−303	1231
210–269	171	2854	2241	3468	1392	176	2608
270–329	141	4246	3208	5284	−1879	−3057	−700
330–389	117	2367	1752	2982	973	−92	2037
390–449	120	3340	2475	4205	411	−1128	1950
450–509	76	3751	2495	5007	−2238	−3474	−1001
510–569	83	1513	1068	1959	870	−34	1775
570–629	68	2384	1600	3168	306	−598	1210
630+	713	2690	2392	2988			

Ninety-five percent confidence intervals are given

Table 5 shows mean annual health system costs are highest at A$5551 in the 601 individuals with 0–29 min of PA per week, consistently reduce in populations with each hour of additional physical activity up to 209 min (A$2390 in 213 individuals), while beyond that show no consistent pattern. Notably, marginal increases in mean health expenditure are observed for populations with 210–329 min in comparison with those from 150 to 209 min. This marginal analysis of health system cost savings evidence per person might be considered as economic support for a threshold between 150 and 209 min. However, in interpreting the health cost implications from the choice of a threshold value, population level total health expenditure effects are a trade-off between the size of the reduction in mean cost per person and the number of people who could realize the reduction by moving from below to above any threshold. As the threshold increases the reduction in mean total cost diminishes, but the number of people who could realize that reduction increases, see Table 6.

**Table 6:** Comparing population health system cost savings across potential PA guideline thresholds

Minutes of activity per week	N	Mean cost per person	Lower limit	Upper limit	Mean difference	Lower limit	Upper limit	Potential annual saving** study population (A$ millions)
		A$						
0–29	601	5551	4890	6213	−2497	−3184	−1811	−1.50
30 or more	2385	3054	2871	3236				
0–59	775	5229	4680	5777	−2258	−2837	−1680	−1.75
60 or more	2211	2970	2786	3155				
0–89	964	4881	4422	5341	−1957	−2454	−1460	−1.89
90 or more	2022	2925	2735	3115				
0–119	1079	4725	4304	5145	−1829	−2292	−1366	−1.97
120 or more	1907	2895	2701	3089				
0–149	1284	4560	4188	4932	−1761	−2182	−1339	−2.26
150 or more	1702	2799	2601	2998				
0–179	1372	4412	4064	4761	−1584	−1989	−1179	−2.17
180 or more	1614	2829	2623	3035				
0–209	1497	4251	3929	4573	−1394	−1782	−1005	−2.09
210 or more	1489	2858	2641	3075				
0–239	1579	4178	3869	4486	−1319	−1700	−938	−2.08
240 or more	1407	2859	2636	3083				

**Number of respondents below the threshold *multiplied by* the mean difference, e.g. for ‘0–29’ versus ‘30 or more’: 601 x (−2497) = −1,500,697, i.e. potential yearly cost savings of A$1.50 million in the study sample

The last column in Table 6 is the number of respondents below the threshold multiplied by the reduction in mean total cost, and represents the total potential cost saving in the sample of 2986 people with health care use and cost data. Potential cost savings are maximised at 150 min per week, providing support for using 150 min physical activity per week as the threshold in maximizing potential population level health system cost savings. That is, comparing total health expenditure in the study population for all potential threshold up to 240 min, Table 6 shows cost savings across the study sample would be maximised with a physical activity threshold of 150 min given a reduction in mean cost of A$1760 per respondent across 1284 respondents maximises potential for health system cost savings at A$2.26 million in the study sample.

These estimates from the second analysis, as with the first analysis, assume that in estimating potential for A$ cost savings across populations annually there are no exogenous confounding effect on cost savings associated with potential differences between those who meet and do not meet guidelines. Potential for confounding (age, sex, socio-economic status) are adjusted in the 3rd set of analyses.

### 3rd analysis of individual level effects under uncertainty with covariate adjustment

For the 3rd and most robust analysis, as in the first two analyses, the outcome of interest is total annual health care cost and the predictor variable of interest is the level of physical activity per week. To enable robust interpretation of this relationship, other variables collected by the Active Lives study, ‘ancillary variables’ in Table 7, were examined as possible confounders in Table 8 partially and Table 9 jointly, while all variables were also considered, but no significant evidence was found, for possible effect modification.

**Table 7:** Active Lives ancillary variables and adjustment for confounding and effect modification

Variable	Possible confounder	Possible effect modifier
Sex	X	X
Age	X	X
Income	X	X
Marital status	X	X
Education	X	X
Work status	X	X
Home ownership	X	X
General health		X
Ability to be physically active		X
Opportunity to be physically active		X
Disabled		X

**Table 8:** Mean difference (≥150 minus <150 min/week) in total cost, with 95% confidence limits

	Mean (A$)	Lower limit (A$)	Upper limit (A$)
No adjustment	−1760	−2182	−1339
Adjusted for sex	−1748	−2171	−1326
Adjusted for age	−1666	−2085	−1247
Adjusted for income	−1572	−2037	−1107
Adjusted for sex and age	−1624	−2045	−1202
Adjusted for sex and income	−1569	−2035	−1104
Adjusted for age and income	−1429	−1891	−968
Adjusted for sex, age and income	−1393	−1857	−928

This table is derived from the data from all respondents, *N* = 2986

**Table 9:** Mean difference (≥150 minus <150 min/week) in total cost, with 95% confidence limits with the four respondents whose total cost exceeded $75,000 removed, *N* = 2982

	Mean (A$)	Lower limit (A$)	Upper limit (A$)
No adjustment	−1473	−1863	−1083
Adjusted for sex	−1460	−1852	−1067
Adjusted for age	−1434	−1821	−1046
Adjusted for income	−1144	−1561	−726
Adjusted for sex and age	−1407	−1797	−1017
Adjusted for sex and income	−1145	−1564	−727
Adjusted for age and income	−1129	−15,434	−714
Adjusted for sex, age and income	−1121	−1538	−704

Hence, the third analysis in considering these variables both adjusts for possible confounders (age, sex, income and other potential socio-economic status measures) in estimating the reduction in total health care cost due to physical activity as well as the potential for effect modification. Potential confounders are appropriately jointly allowed for with multivariate regression analysis in Table 8 to adjusts for exogenous population differences in estimating cost impacts of physical activity levels.

The bottom line is that after adjusting for sex, age and income as covariates health care costs are lower by $1393 per person–year for those who meet PA guidelines. While overall this is $367 lower than the $1760 estimate without any adjustment, partial analysis shows that income then age and sex had the greatest attribution in leading to this combined reduction.

The estimates in Table 8 include the data from all respondents, allowing for the impact of high-cost respondents, where four individuals had costs greater than A$75,000, three of whom were in the population who did not meet the guidelines. Nevertheless, to allow the most robust analysis as well as a conservative base case analysis, the cost estimates in the population who do not meet guidelines and more importantly the potential incremental cost saving expected from shifting populations from those who do not meet guidelines to those who do can be significantly improved in terms of stability with removal of the four individuals with costs greater than A$75,000. Table 9 is the same as Table 8, with the four respondents with total costs greater than A$75,000 removed. The presence of these high cost respondents skew the distribution and raise concerns regarding the reliability of the 95% confidence intervals.

Removing the four high-cost respondents from the sample, the incremental cost difference with covariate adjustment for sex, age and income between populations meeting and not meeting guidelines reduces by A$272 per person–year from A$1393 per person–year to A$1121 (95% CI A$704, A$1538). For analyses where the four high-cost responders were removed, this also represents a A$352 reduction compared to the unadjusted analysis, where incremental costs were estimated as A$1473 per person–year. Although removing the high cost respondents lowers the reduction in health care cost due to physical activity, the width of the confidence intervals remains mostly unchanged, demonstrating the robustness of the results.

The A$1121 estimate, adjusting for sex, age and income and removing four high-cost respondents, provides the most conservative base case estimate of overall potential cost savings. Overall, while confounder adjustment alone reduces the incremental cost saving on average by 20.9% from A$1760 to A$1393 per person–year, this increases to a 36.3% reduction, or A$1121 per person–year additionally allowing for a conservative robust base case analysis removing four high-cost respondents.

Table 10 contains the population level impact if health promotion and whole of government strategies in support of PA guidelines enabled shifting SA adult populations who currently undertake 0–149 min of physical activity to have physical activity reflecting that of populations who undertake 150+ minutes at a population level.

**Table 10:** Third analysis of potential for health system cost saving from adults meeting PA guidelines with covariate adjustment

SA adult population estimate June 2018	1,369,751
Survey population proportion not meeting guideline	42.12%
Estimated SA population not meeting guideline	576,954
Lower health cost per adult meeting vs not meeting PA guideline excluding the four high cost respondents	A$1121
Potential annual lower net health care cost	A$646,765,434

Hence, the confounder adjusted analysis with a conservative base case suggests if SA adult populations who currently undertake 0–149 min of physical activity shifted to having physical activity reflecting that of populations who undertake 150+ minutes then net government health expenditure annually in SA has potential to reduce by $646 million annually. If high cost respondents are included, this increases to $803 million annually.

In terms of more realistic intra (extra population) and/or infra (nudge strategies across whole population) marginal effects multivariate joint covariate adjusted analysis from individual person data suggests, with removal of high cost tails:

(i) An absolute 5% intramarginal reduction in the proportion of the population who do not meet PA guidelines, i.e. from 42.12% to 37.12%, which equates to potential cost savings of 0.05/0.4212 × A$646 million = A$76.7 million per year.
(ii) A relative 5% intramarginal reduction in the proportion of the population who do not meet guidelines – from 42.12% to 40.06%, which equates to potential health system cost savings of 0.05 × 576,954 × A$1121 = A$32.3 million per year.
(iii) A relative inframarginal reduction in health care costs per person across the population who do not meet guidelines of 5% towards the level of that of populations who do meet guidelines which equates to potential health system cost savings of 0.05 × A$646 million = A$32.3 million per year.

### Consistent covariate adjusted wellbeing analysis for joint cost and effect consideration

To enable robust health economic analysis costs and effects need to be jointly considered, and more generally robust decision making requires coverage and comparability principles in evaluating such joint consideration of costs and effects should be met. Mirroring multivariate age, sex and income adjusted covariate analysis of costs, consistent multivariate covariate adjustment should also be considered for the Active Lives South Australia study subjective wellbeing assessed as with UK Sports England annual survey questions for overall wellbeing and disaggregated components for:

(i) Satisfied with life nowadays
(ii) Things you do in life are worthwhile
(iii) How happy did you feel yesterday
(iv) How anxious did you feel yesterday

Table 11 shows that in each case odds ratios for good outcomes (scores of 7 to 10 on first 3 and 0–3 on fourth) on these subjective wellbeing measures statistically significantly improved with meeting guidelines with unadjusted or covariate adjusted analysis. Combining these to identify overall good wellbeing (scoring 7 to 10 on 1st 3 and 0–3 on 4th), the odds of scoring well for overall wellbeing also significantly improved. Overall covariate adjustment maintained both significant effects and their extent.

**Table 11:** Covariate adjusted odds ratio overall wellbeing scored as being ‘good’ or not ‘good’ in populations meeting vs not meeting physical activity guidelines (≥150 versus <150 min/week)

	Odds ratio (95% C.I.)	*p* value
No adjustment	1.82 (1.55, 2.13)	<0.0001
Adjusted for sex (*p* = 0.400)*	1.81 (1.55, 2.12)	<0.0001
Adjusted for age (*p* < 0.0001)	2.00 (1.71, 2.35)	<0.0001
Adjusted for income (*p* = 0.209)	1.89 (1.57, 2.26)	<0.0001
Adjusted for sex (*p* = 0.914) and age (*p* < 0.0001)	2.00 (1.70, 2.35)	<0.0001
Adjusted for sex (*p* = 0.191) and income (*p* = 0.235)	1.87 (1.56, 2.25)	<0.0001
Adjusted for age (*p* < 0.0001) and income (*p* < 0.0001)	1.97 (1.64, 2.37)	<0.0001
Adjusted for sex (*p* = 0.949), age (*p* < 0.0001), and income (*p* < 0.0001)	1.97 (1.64, 2.37)	<0.0001

*p values in the first column are for the association between the covariate and scoring ‘good’ for overall wellbeing

For overall wellbeing, both unadjusted and covariate adjusted analysis had highly significant ORs for populations meeting relative to not meeting PA guidelines of scoring highly for overall wellbeing. Indeed, combined age sex and income covariate adjusted ORs for overall wellbeing increased somewhat compared to unadjusted analysis (1.97 vs 1.82). The last column of Table 11 shows the extent of the odds ratio benefit is highly significant (<0.0001) regardless of what combination of age, sex and income factors are adjusted for. The significance of each variable adjusted for in covariate analysis is indicated in the first column where age and income are highly significant and should be adjusted for in each case, while addition of sex is shown to make no difference with three significant figures to the extent (1.97) or CI (1.64, 2.37) of covariate adjusted analysis. Similar patterns of significantly higher odds ratios for having good wellbeing outcomes in populations meeting vs not meeting PA guidelines and those odds ratios being maintained with covariate adjustment for age, sex and income were found for each of the disaggregated components of wellbeing, summarised in Table 12.

**Table 12:** Covariate adjusted odds ratios for subjective wellbeing components in populations meeting vs not meeting physical activity guidelines (≥150 versus <150 min/week)

	Odds ratio (95% C.I.)	*p* value
Score 7+ ‘Satisfied with life nowadays’ no adjust	3.00 (2.51, 3.59)	<0.0001
Adjusted for sex (*p* = 0.850), age (*p* < 0.0001), and income (*p* < 0.0001)	3.04 (2.47, 3.74)	<0.0001
Score 7+ ‘Things you do in life are worthwhile’ no adjust	2.31 (1.89, 2.83)	<0.0001
Adjusted for sex (*p* = 0.0039), age (*p* < 0.0001), and income (*p* < 0.0001)	2.35 (1.86, 2.98)	<0.0001
Score 7+ ‘How happy did you feel’ no adjust	2.21 (1.86, 2.62)	<0.0001
Adjusted for sex (*p* = 0.863), age (*p* < 0.0001), and income (*p* < 0.0001)	2.40 (1.97, 2.93)	<0.0001
Score 0–3 ‘How anxious did you feel?’ no adjust	1.45 (1.25, 1.69)	<0.0001
Adjusted for sex (*p* = 0.014), age (*p* < 0.0001), and income (*p* = 0.009)	1.42 (1.19, 1.69)	<0.0001

*p-values in the first column are for the association between the covariate and scoring

The last column of Table 12 shows the extent of the odds ratio benefit is highly significant (0.0001 or less) with or without age, sex and income adjustment for each component. The significance of each variable adjusted for in covariate analysis is indicated in the first column where age and income are highly significant and should be adjusted for in each case while sex was also significant for ‘how anxious did you feel’ and ‘things you do in life are worthwhile’.

These findings for overall wellbeing and across its decomposed components for the general maintaining of the significance and extent of subjective wellbeing benefits from meeting PA guidelines with age, sex and income covariate adjustment are consistent with that in main study with age, sex and SEIFA covariate adjustment (Active Lives South Australia 2019). Hence, more generally triangulation across these findings produce mutually supportive evidence of statistically and clinically significant wellbeing benefits from meeting PA guidelines whether unadjusted or with age, sex and income or age, sex and SEIFA covariate adjustment, which in turn provide evidence for and support findings of lower health care utilization and costs in meeting PA guidelines.

## Discussion

The Active Lives South Australia study and survey undertaken in April 2019 enabled comparison of self-reported measures in almost 3000 adults who meet or do not meet physical activity guidelines in undertaking 0–149 min or 150 min or more of physical activity per week. The main study estimated that 42.12% of the South Australian population do not currently meet physical activity guidelines and that adult populations relative to those not meeting guidelines had associated improved wellbeing, individual development, social connectedness and social capital. Trends were also found for adult populations meeting guidelines having lower probability of visits in the last year to GP, specialist doctor or other health professional or hospital inpatient admissions, outpatient clinic and emergency department visits, while higher probability of using dentists.

pt?>The health economic analyses presented in this paper aimed to extend the main study analysis to start to inform the need for active live health promotion policy initiatives through robustly estimating potential population level health system cost savings from shifting adult populations from not meeting PA guidelines to meeting guidelines. Findings to that end have been reported across increasingly robust and instructive analysis. The first analysis used individual data in estimating odds of services use, overall resource use and associated cost differences in dichotomous populations meeting and not meeting PA guidelines allowing for gamma distributions to fit skewed cost data while not adjusting for potential effects of differences in covariates between these populations. Interestingly, while odds, use and costs for hospital inpatient, outpatient and ED, specialist doctor and GP use were all significantly lower in populations meeting PA guidelines, odds of using dentists were higher, alongside non-significant trends for higher net dental use and costs. The higher proportion of population using dental services in the population meeting PA guidelines were mitigated in estimating total dental costs per person–year by their lower average use per person in those using dental services (see Tables 1 and 2), consistent with those who meet PA guidelines engaging in more preventative activities generally. That is, populations meeting PA guidelines proportionately having higher preventive utilisation of dental services while not higher acute dental services. Nevertheless, further research and particularly research on the breakdown of type of dental, and perhaps also GP service use by preventative vs acute services would be required to establish evidence for that or other potential explanations.

The second set of analyses considered marginal rather than dichotomous consideration of physical activity levels to consider whether the physical activity guideline threshold of 150 min per week is supported by potential health care cost savings and more generally estimating marginal effects across levels of PA per week. This second analysis established health economic support for current adult physical activity guidelines of 150 min per week with the greatest population cost saving potential from meeting guidelines at that threshold level from 30 min intervals considered (see Table 6). The third and most robust analysis uses individual data to estimate cost differences under uncertainty in comparisons between populations meeting and not meeting guidelines while undertaking multivariate adjustment for potential covariates. Intra and inframarginal population impacts of strategies across populations and sensitivity analysis to consider the impacts of removing high cost respondents (greater than A$75000) to allow robust modelling of a conservative estimate of cost savings were also undertaken. Results comparing covariate adjusted and unadjusted analysis of potential cost savings and marginal population impacts as well as sensitivity analysis removing impacts in tails for high cost respondents are summarised in Table 13.

**Table 13:** Summary of individual and population level potential annual A$ health care cost savings if strategies shift populations from not meeting to meeting PA guidelines

		Per adult	Whole population level*	Marginal shift	
				Absolute 5% population	5% of those not meeting
No covariate adjustment		A$	A$ million	A$ million	A$ million
1st analysis (individual, continuous)	High cost respondents included	1760	1015	120.5	50.8
Conservative	High cost respondents removed	1473	850	100.9	42.9
Covariate adjustment		$	$ million	$ million	$ million
3rd analysis (individual, continuous)	High cost respondents included	1393	804	95.4	40.2
3rd analysis (individual, continuous)	High cost respondents removed	1121	647	76.8	32.3

*42.12% of adult population not meeting guidelines or 576,954 of SA adult population of 1,369,751 at June 2018

Comparison between results from these increasingly robust levels of analysis and sensitivity analysis in Table 13 is instructive in seeing

(i) The impact of allowing for covariate adjustment and;
(ii) The impact of removing high cost respondents in comparison between sensitivity and base case analyses.

Overall, allowing for covariate adjustment mitigated somewhat the findings of potential for health system cost savings in meeting vs not meeting PA guidelines for individuals, at a population level and in terms of potential for cost savings with more 5% marginal effects without adjustment and to about the same extent as undertaking conservative base case analysis with removal of high cost respondents. Nevertheless, for the most robust 3rd analysis with covariate adjustment, even under the most conservative model with high-cost respondents removed, the potential annual cost saving remains more than A$1000 per adult (A$1121) from meeting vs not meeting PA guidelines and A$646 million annually if the whole population were shifted. The potential cost saving to the SA health system from more achievable 5% marginal shifts with health promotion strategies in the population under this conservative assumption ignoring high-cost respondents remain more than A$30 million annually.

More generally advantages of analysing individual-level data arise:

(i) In the second analysis in providing evidence in support of using 150 min per week as the threshold;
(ii) In the first and third analyses enabling removal of high cost outliers to undertake conservative sensitivity analysis;
(iii) In the third analysis adjusting the estimates of health care cost saving in meeting the threshold for the confounders of sex, age and socio-economic status; and
(iv) Analysis of potential effect modification providing evidence that there is no easily identifiable sub-group that would benefit most from meeting the threshold, or equivalently all populations benefit from meeting the PA guidelines.

### Study potential strengths and weaknesses

#### Causality considerations

The results of analysis of costs and effects in this study have been based on associations between levels of PA or meeting vs not meeting PA guidelines and health use, cost and wellbeing measures observed in cross-sectional analysis, and with multivariate covariate adjustment in the most robust sets of analysis. Nevertheless, whether covariate adjusted differences in health expenditure and effects observed cross-sectionally across populations represent what could be expected if strategies change PA behaviours, and meeting of guidelines over time naturally also needs consideration of causality and its direction. Considering the potential for reverse causality (improved wellbeing leads to meeting PA guidelines) or potential for health related (e.g chronic disease) explanations where chronic disease health improvement leads longitudinally to change in behaviour in meeting PA guidelines, it is important to note that analysis, while cross-sectional, found:

1. Covariate adjustment only marginally mitigated significant levels of cost saving and maintained (or slightly increased) significant wellbeing effects;
2. No effect modification on cost savings from meeting vs not meting guidelines was evident for health-related variables and in particular general health, ability of opportunity to be physically active or disability variables, with such lack of effect modification providing cross sectional evidence that do not support changes in those variables explaining cost saving in longitudinal analysis.

Furthermore, recent literature provides broader supportive evidence of positive health effects from meeting guidelines in review of both:

3. Physical activity evidence alone underlying adult integrated movement guidelines (Ross et al. 2020) in meeting versus not meeting physical activity guidelines (18 to 64 and 65 and older with 150 min of moderate to vigorous PA per week and resistance bone/muscle strengthening twice week and including some balancing exercises or activities); and
4. Meeting 24-h integrated movement guidelines (joint PA, sedentary – less than 8 h of screen-time and sleep behaviours – 7–9 h for 18–64 years and 7–8 h for over 65 populations) in evidence review underlying adult integrated movement guidelines (Ross et al. 2020).

In relation to 3 and 4, evidence review from 24-h integrated movement guidelines (IMGs) for adults (Ross et al. 2020) indicate meeting 150 min of moderate to vigorous PA as part of IMGs was associated with better all-cause mortality, adiposity and cardiometabolic biomarkers and overall health generally.

More generally, compositionally ‘24-h movement behavior composition was associated with all-cause mortality, adiposity, and cardiometabolic biomarkers.’ Triangulating associations across the evidence base for all health behaviours and outcomes, Ross et al. (2020) review evidence found that ‘health would improve if time was reallocated into moderate to vigorous physical activity (MVPA) or if time were taken out of sedentary behaviour and reallocated into sleep or light PA. Health would worsen if time was taken out of MVPA, irrespective of what other movement behaviour MVPA was reallocated out of, or into.’ Further, Ross et al. (2020) note ‘These findings support the notion that the intensity of movement across the entire 24-h day matters and that recommendations for sleep, sedentary, and physical activity can be combined into a single public health guideline’ and that ‘Following these Guidelines may be challenging at times; progressing towards any of the Guideline targets will result in some health benefits.’

#### Key need for future longitudinal research across integrated movement behaviours

24-h integrated movement guidelines in combining and appropriately jointly considering the interrelated nature of time spent in physical activity, sedentary behaviour and sleep across any 24-h period come closest of current public health measures to overcome the perils of partialisation in representing lifestyle and key habit forming behaviours. To best model or estimate health effects of strategies in any given population of shifting the composition of movement behaviours is best undertaken with *compositional isotemporal substitution* methods (Dumuid et al. 2018). These methods can be used to model the effects of interventions, or to design interventions to optimise outcomes, for example, by nudging participants to make optimal reallocation decisions across time constrained joint integrated movement and lifestyle behaviours. Many of the limitations of previously partialized or siloed consideration of individual behaviours in either not considering unintended consequences of just focusing on one behaviour or failing to account for double or multiple counting of benefits from related time constrained lifestyle behaviours are overcome with their joint consideration with 24-h integrated movement behaviours and their composition (PA, sleep and sedentary time) and the multiple health effects that they can influence. Naturally, such longitudinal research on IM behaviour change is supported as valuable in conclusively answering and put to bed the potential for reverse causality explaining wellbeing and cost benefits from meeting PA and IM guidelines.

These benefits of such research are further reinforced by the coverage and continuity of integrated movement guidelines across the life-course from early childhood in children under 5 (Tremblay et al. 2017; Okely et al. 2017; World Health Organisation 2019), children and youth from 5 to under 18 (Tremblay et al. 2016), as well as most recently adults 18 to under 65 and 65 and older (Ross et al. 2020). The combined evidence of health benefits from meeting 24-h integrated movement guidelines in improving health generally and all-cause mortality, adiposity and cardiometabolic biomarkers in particular across the life-course in reviews underlying these guidelines starts to clarify the potential of community-based health promotion strategies in support of integrated movement guidelines and associated behaviour compositions.

Our study results for reduced health care utilisation and costs in meeting PA guidelines in adult populations broadly support wellbeing and health effects promoted with the adult IMGs and their evidence review (Ross et al. 2020). Nevertheless, evidence of health system resource use and cost benefits from meeting PA guidelines associated with improvement in wellbeing in this study, such as evidence in IMG reviews of health benefits, point to need for longitudinal research to confirm potential cost savings where health promotion strategies improve PA and integrated movement behaviours. For example, environmental and whole of government strategies with walking and bike paths and linking public transport to use of community spaces (e.g. libraries, piazzas, national parks, community gardens, public swimming pools, beaches, etc.) where they have community ownership can be expected to support meeting of integrated movement guidelines and behaviours more generally across the life-course. That is, with community ownership they would be expected to benefit active and successful ageing of adult populations in the community in improving their 24-h integrated movement behaviours and their associated all-cause mortality, adiposity and cardiometabolic biomarkers, but also active and successful development and habit forming in early childhood and youth populations and their lifetime integrated movement behaviour trajectories and associated all-cause mortality, adiposity and cardiometabolic biomarkers. Importantly, this health economic analysis of the Active Lives South Australia study, alongside improved wellbeing, meeting physical activity guidelines alone is associated with significantly lower health care costs in adult populations. Indeed, meeting versus not meeting PA guidelines alone (150+ vs less than 150 min of PA per week) has been estimated to have A$1393 lower costs after jointly adjusting for age, sex and income covariates pointing to potential for health system cost savings where strategies can shift community behaviours.

#### Policy implications – critical need for effective health promotion of active communities

In general, the extent to which potential long term health system cost savings at a population level from meeting PA guidelines are expected to arise in practice depends on the effectiveness, community ownership and associated population multiplier effects of community health promotion strategies (Eckermann et al. 2014; Eckermann and McCaffrey 2017; Hawe and Ghali 2008, Hawe et al. 2009; Shiell and Hawe 1996, Shiell et al. 2008, Shiell and Jackson 2018; Rychetnik et al. 2002; Zaza et al. 2005). It is important to recognise that effective health promotion strategies and multiplier effects are associated with positive community network effects, social connections and social capital formation. Health promotion programs that in the long-term increase community connectedness and build social capital are key to the effectiveness of health promotion programs. In that respect the Active Lives South Australia study has shown significant positive relationships in populations meeting PA guidelines with higher levels of social capital and community connectedness. Hence, strategies that improve population compliance with PA and integrated movement guidelines *apriori* are also expected to contribute towards social capital formation and positive multiplier effects.

In ageing adult populations health promotion strategies that have been effective in promotion of active lives in communities and populations internationally are whole of government strategies for Age and Dementia friendly communities. Such strategies include age and dementia friendly transport systems, taxi drivers, shops, walking paths, community gardens and more generally amenity to and community programs in support of use of public spaces such as libraries and parks (Eckermann 2017 Chap 12; Eckermann et al. 2019). Activities in public places should also consider cultural appropriateness for any given community setting with, for example, socially and culturally appropriate activities in different community settings such as tai chi, boules (lawn bowls, bocce or petanque), salsa dancing, community gardening or community food gathering and foraging, communal eating clubs or more generally cooking and/or eating together. Such environmental design strategies could also involve supporting carer and living arrangements of ageing populations in the community with flexible housing and more generally modified homes and communal living arrangements such as co-housing, granny flats, laneway housing, naturally occurring retirement communities and virtual retirement villages (Newton 2015). In each case, as with design of dementia friendly age care such strategies, while having potential benefits for populations in being active and meeting physical activity and integrated movement guidelines, they do not necessarily incur any net additional cost and indeed can reduce costs. That is, where they are effective in enabling more active healthier populations with community ownership, the potential arises for such strategies to be inexpensive relative to health care cost savings.

In over 65 populations even greater potential cost savings arise from avoiding age care costs (Kalache 2013; Eckermann 2017 Chap 12; Eckermann et al. 2019). In that respect, it should be noted that the direct health and residential care costs of people living with dementia in South Australian residential aged care are estimated to be A$88,000 per resident per year, while A$12,962 less with clustered residential versus larger institutional style care after adjusting for resident- and facility-related factors (Dyer et al. 2018; Gnanamanickam et al. 2018). Importantly in undertaking reform of age and health care environments towards budget constrained successful ageing these clustered, domestic scale models of residential aged care have also been shown to have significantly higher quality of life for residents than institutional aged care (EQ-5D-5L score greater by 0.107, *p* = 0.008), while significantly reducing psychotropic medication (OR = 0.24) and all medication use (OR = 0.48) as well as hospitalisations and emergency department presentations and costs (Harrison et al. 2018a, b; Dyer et al. 2018).

More generally, as the WHO age friendly cities guidelines (World Health Organisation 2007) and Alzheimer’s Disease International (2016) dementia friendly guidelines highlight and Alexandre Kalache emphasised in his case study and recommendations for South Australia, facing the challenge of baby boomer ageing there is an imperative to invest in age friendly communities to support successful ageing of baby boomer populations and reduce health and age care costs. In the absence of physically, mentally and socially active baby boomer populations South Australia, Australia and indeed the World more generally will face burgeoning health and aged care costs while not meeting baby boomer preferences for active successful ageing, what Kalache (2013) coined ‘Gerentolescence’ – the adolescence of old age.

Reforms in caring for aging populations, including those with dementia, are increasingly being called for internationally in the context of a threefold increase in numbers associated with ageing of baby boomers projected from 2010 to 2050 (World Health Organisation and Alzheimer’s Disease International 2012; World Health Organisation 2015; Alzheimer’s Disease International 2016; Eckermann 2017, Chap 12; Eckermann et al. 2019). What has emerged as clear in addressing this challenge within budget constraints is the importance of environmental and whole of governments solutions and the right policy balance of community and care based environmental strategies to enable budget constrained successful ageing across health and aged care systems (World Health Organisation 2002, 2007, 2017; Davis et al. 2009; Kalache 2013; Eckermann 2017, Chap 12; Phillipson et al. 2018; Eckermann et al. 2019). In practice, supporting low cost whole of government approaches and avoiding high cost of health and aged care through the creation of age and dementia friendly communities is the most effective and efficient strategy.

Australia, as with other developed countries, has suffered from a chronic underfunding of community health promotion (Jackson and Shiell 2017). We have also failed to capitalise on the potential for the built environment to promote opportunities for our older populations to enable active ageing (World Health Organisation 2015). All of these point to a primary need for whole of government health and age care strategies and regulatory reform in creating age and dementia friendly environments to jointly address health and age care needs in research and practice (Eckermann 2017, Chap 12; Eckermann et al. 2019).

Furthermore, such environmental and whole of government strategies for age and dementia friendly communities are more generally supportive of social connections and social capital active communities across the lifecycle. In early childhood (Okely et al. 2017), youth (Tremblay et al. 2016) and adult 18–64 and 65 and older populations (Ross et al. 2020) the same environmental and whole of government policies are key to supporting 24-h integrated movement behaviours (joint physical activity, sedentary behaviour and sleep). These are in turn the best public health indicators for positive habits and lifestyle behaviours from early years across the life cycle. Child populations meeting IMGs are associated with better body composition, cardiorespiratory and musculoskeletal fitness, cardiovascular and metabolic health, academic achievement and cognition, mental health and quality of life, emotional regulation, and pro-social behaviours in childhood and across the life cycle (Dumuid et al. 2018; Okely et al. 2017; Tremblay et al. 2016; Katzmarzyk et al. 2013; Walsh et al. 2018; Saunders et al. 2016; Roman-Viñas et al. 2016). An evidence review of the IMG for Adult populations (Ross et al. 2020) similarly found meeting 150 min of moderate to vigorous PA per week was associated with better all-cause mortality, adiposity and cardiometabolic biomarkers particularly and overall health generally. While across IMB behaviours compositionally ‘24-h movement behaviour composition was associated with all-cause mortality, adiposity, and cardiometabolic biomarkers’ (Ross et al. 2020). Hence, creating environments and whole of government policies for active communities is more generally supportive of active lives in all adult as well as young and ageing populations. The importance of social connectedness and social capital formation in creating active communities and populations is also supported by evidence in the Active Lives survey and study itself. The Active Lives survey found consistent positive association between meeting PA guidelines (having 150+ versus 0–149 min of PA per week) and community connectedness, trust, identity, social capital formation, individual development, subjective health status and wellbeing (Active Lives South Australia 2019).

## Conclusion

Health Economic analysis of the Active Lives South Australia study has shown adults meeting physical activity (PA) guidelines with 150 min or more of PA per week are associated with statistically significantly lower overall utilisation and costs of public health care use as well as improved overall wellbeing and was particularly evidence for acute care components (hospital admissions, outpatient and ED, clinical specialists). That was the case with and without jointly adjusting in multivariate analysis for age, sex and income covariates. Treating PA as a continuous variable marginal analysis provides support for current guidelines of 150 min PA per week in providing the greatest potential for health cost saving at a population level.

The most robust multivariate covariate adjusted analysis found lower health care costs of A$1393 per patient year in meeting guidelines. This translates to cross sectional evidence of a potential health system cost saving in South Australia of A$804 million annually if the 42.12% of adults not meeting guidelines could be shifted to meeting guidelines. There was no significant effect modification across any covariates providing broad support for health system cost saving potential of health promotion strategies in support of meeting adult PA guidelines to improve wellbeing.

Nevertheless, actually realising savings requires health promotion policies with community ownership that enable shifting populations level behaviours, where more realistically changes in individuals behaviours are expected to arise at the margins for shifting populations (intramarginal) and behaviours (inframarginal), and should consider where PA changes arise from, following integrated movement guidelines.

Policy implications point to the cost saving potential of strategies for health promotion of physical activity and integrated movement guidelines and value of further research on longitudinal evidence of integrated movement behaviours and their health, health care utilisation and cost implications. Analysis of community ownership and multiplier effects that can arise with such health promotion strategies where effective are key to robustly assessing these impacts in each case. These research studies are particularly important currently given the need to find effective and ideally cost-saving strategies to help populations and health systems recover from, wellbeing challenges and highly budget and activity constrained environments that have arisen globally with the COVID-19 pandemic.

## Data Availability

All methods in this paper are illustrated with data from the active lives South Australia study.

## Footnotes

1 Constructing a confidence interval or testing the statistical significance of these odds ratios is also simply undertaken given the natural logarithm relationship with odds ratios. Hence the standard error (SE) for the natural logarithm of the OR is simply the square root of the sum of the reciprocal for number of events and non-events in the compared populations. The lower and upper 95% CI around the natural logarithm of the mean OR simply subtract and add 1.96 of these standard errors. Taking the exponential of these natural logarithms of the mean OR and 95% CI enables the mean and 95% CI of the OR to be reported. Hence, for example, for GP services with a mean OR of 0.48 ((1554/186)/(1194/65)), ln (0.48) = −0.733 has SE = $\sqrt{\frac{1}{1554}+\frac{1}{186}+\frac{1}{1194}+\frac{1}{65}}$ = 0.0796 and hence 95% CI is exp.(−0.733±1.96(0.0796)) = (0.41, 0.56).
